# Genome-wide association analysis of canine T zone lymphoma identifies link to hypothyroidism and a shared association with mast-cell tumors

**DOI:** 10.1186/s12864-020-06872-9

**Published:** 2020-07-06

**Authors:** Julia D. Labadie, Ingegerd Elvers, Heather Spencer Feigelson, Sheryl Magzamen, Janna Yoshimoto, Jeremy Dossey, Robert Burnett, Anne C. Avery

**Affiliations:** 1 Public Health Sciences Division, Fred Hutchinson Cancer Research Center, Seattle, WA USA; 2 Department of Environmental and Radiological Health Sciences, College of Veterinary Medicine and Biomedical Sciences, Colorado State University, Fort Collins, CO USA; 3 Department of Medical Biochemistry and Microbiology, Uppsala University, Broad Institute of MIT and Harvard, Cambridge, Massachusetts and Science for Life Laboratory, Uppsala, Sweden; 4 Kaiser Permanente, Institute for Health Research, Aurora, CO USA; 5 Department of Microbiology, Immunology and Pathology, College of Veterinary Medicine and Biomedical Sciences, Colorado State University, Fort Collins, CO USA

**Keywords:** Lymphoma, Leukemia, Genetics, Dog, Epidemiology

## Abstract

**Background:**

T zone lymphoma (TZL), a histologic variant of peripheral T cell lymphoma, represents about 12% of all canine lymphomas. Golden Retrievers appear predisposed, representing over 40% of TZL cases. Prior research found that asymptomatic aged Golden Retrievers frequently have populations of T zone-like cells (phenotypically identical to TZL) of undetermined significance (TZUS), potentially representing a pre-clinical state. These findings suggest a genetic risk factor for this disease and caused us to investigate potential genes of interest using a genome-wide association study of privately-owned U.S. Golden Retrievers.

**Results:**

Dogs were categorized as TZL (*n* = 95), TZUS (*n* = 142), or control (*n* = 101) using flow cytometry and genotyped using the Illumina CanineHD BeadChip. Using a mixed linear model adjusting for population stratification, we found association with genome-wide significance in regions on chromosomes 8 and 14. The chromosome 14 peak included four SNPs (Odds Ratio = 1.18–1.19, *p* = .3 × 10^− 5^–5.1 × 10^− 5^) near three hyaluronidase genes (*SPAM1, HYAL4,* and *HYALP1*). Targeted resequencing of this region using a custom sequence capture array identified missense mutations in all three genes; the variant in *SPAM1* was predicted to be damaging. These mutations were also associated with risk for mast cell tumors among Golden Retrievers in an unrelated study. The chromosome 8 peak contained 7 SNPs (Odds Ratio = 1.24–1.42, *p* = 2.7 × 10^− 7^–7.5 × 10^− 5^) near genes involved in thyroid hormone regulation (*DIO2* and *TSHR*). A prior study from our laboratory found hypothyroidism is inversely associated with TZL risk. No coding mutations were found with targeted resequencing but identified variants may play a regulatory role for all or some of the genes.

**Conclusions:**

The pathogenesis of canine TZL may be related to hyaluronan breakdown and subsequent production of pro-inflammatory and pro-oncogenic byproducts. The association on chromosome 8 may indicate thyroid hormone is involved in TZL development, consistent with findings from a previous study evaluating epidemiologic risk factors for TZL. Future work is needed to elucidate these mechanisms.

## Background

T zone lymphoma (TZL), a histologic variant of peripheral T cell lymphoma (PTCL), accounts for about 12% of all canine lymphomas [1, 2] but is almost never seen in human patients. In dogs, this disease follows an indolent course with average survival of > 2 years independent of treatment, compared to < 1 year with most other lymphoma subtypes [3–5]. TZL can be readily diagnosed by histopathology or by flow cytometric identification of a homogeneous expansion of T cells lacking expression of CD45, a pan-leukocyte surface marker [3, 6, 7]. Previously, we observed that > 30% of Golden Retrievers without lymphocytosis or lymphadenopathy have T cells phenotypically similar (lacking CD45 expression) to TZL in their blood [8]; as we are unsure of the clinical relevance of this finding, we have adopted the term T zone-like cells of undetermined significance (TZUS) for these dogs [9]. We hypothesize that TZUS may represent a pre-clinical state that could undergo neoplastic transformation and progress to overt TZL.

Few studies have investigated the pathogenesis of canine TZL. We recently reported that both hypothyroidism and omega-3 supplementation are associated with decreased odds of TZL [9]. It has also been noted that over 40% of TZL cases are Golden Retrievers [3]. This finding suggests a genetic predisposition for TZL and caused us to pursue a study to identify potential pathways of interest. To date, no studies have agnostically evaluated germline risk for PTCL in dogs or humans.

The objective of this study was to identify genetic risk factors for canine TZL using a genome-wide association study (GWAS) and subsequent targeted sequencing. This aim of this study is to provide insight into the etiology and underlying risk for developing this disease.

## Results

The source population included 95 TZL cases (ages 7–14 years), 142 TZUS dogs > 9 years old (dogs with no clinical signs of TZL, but > 1% of T cells were CD5^+^CD45^−^), and 101 control dogs > 9 years old (dogs with no clinical signs of TZL and no CD5^+^CD45^−^ T cells). Sixteen dogs were removed due to low genotyping rate (< 97.5%; 7 TZL, 5 TZUS, 4 controls) and 6 were removed due to suspected European origin (2 TZUS, 4 controls). After quality filtering a final dataset of 267 dogs (79 TZL, 108 TZUS, 80 controls), and 110,405 single nucleotide polymorphism (SNPs) were used for association analyses.

### TZUS and controls indistinguishable by GWAS

**Fig. 1:**
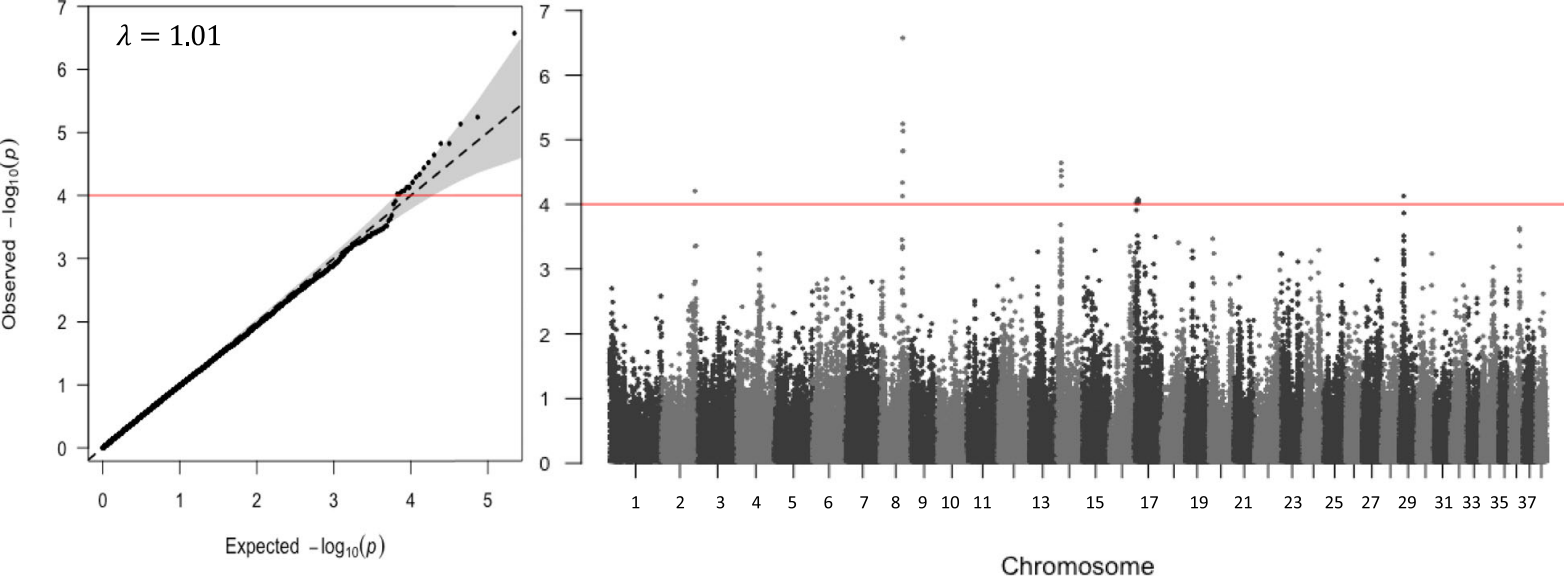
GWA for TZL cases vs. combined reference (TZUS + controls). Left, QQ-plot demonstrating observed *p*-values deviate from the expected at a significance level of *p* < 10^− 4^. Shaded area indicates 95% confidence interval. Right, Manhattan plot showing peaks that are significantly associated with TZL at a genome-wide level of *p <* 10^− 4^

When the combined TZL and TZUS group was compared to controls, no *p*-values were outside the 95% confidence interval threshold on the quantile-quantile (QQ)-plot (Additional file 1 A). In contrast, when TZL were compared to the combined TZUS and control group, a group of SNPs significantly deviated from the expected distribution (Fig. 1). Supporting this, pairwise GWAS of TZL versus controls and TZL versus TZUS had suggestive associations for this group of SNPs, despite none of the *p*-values falling outside the 95% confidence interval (CI) on the QQ-plot (Additional file 1 B and C). This implies TZUS and controls are similar, and the enhanced power from combining them as a reference group allows those SNPs to reach genome-wide significance. In contrast, the TZUS versus control comparison did not share any suggestive SNPs with the TZL versus control comparison, as would be expected if TZL and TZUS were similar. We thus chose to combine TZUS and controls for our main analysis and will reference it as the “TZL versus all” comparison for the remainder of the paper.

### Top peak is near thyroid stimulating hormone receptor locus

**Fig. 2:**
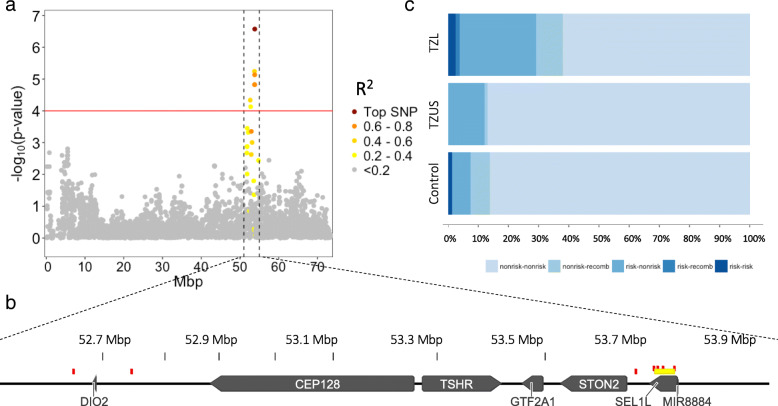
Close-up of the chromosome 8 peak. **a** R^2^ from top SNP (BICF2P948919) is depicted to show LD structure. **b** Close-up view of the genes located in the region with R^2^ > 0.2. All associated SNPs are depicted in red; the haplotype block containing the top 4 SNPs is highlighted in yellow. **c** Haplotype block containing the 4 associated SNPs (BICF2P1080535, BICF2P1048848, BICF2P184533, and BICF2P948919). The risk haplotype was TAGG and non-risk was CGAA. Dogs were considered recombined if neither combination was present

The strongest GWAS peak contained seven SNPs on chromosome 8 from 52,650,576–53,818,371 bp (Fig. 1; Table 1). The associated allele for these SNPs was present in about 16% of TZL (range 15–25%) compared to 6% of the reference group (range 4–12%). The top SNP (BICF2P948919; Odds Ratio [OR] = 1.39, *p* = 2.66 × 10^− 7^) was located at 53,818,371 bp and was in strong linkage disequilibrium (LD) (R^2^ > 0.7) with three significantly associated SNPs in that region and moderate LD (R^2^ 0.25–0.6) with the other three significantly associated SNPs (Fig. 2). Using the PLINK clumping analysis, we determined that the four SNPs in strong LD (including the top SNP) formed one haplotype block, and the remaining three SNPs were not in strong enough LD with any other SNPs to form blocks. The *p*-values for all seven associated SNPs on chromosome 8 were non-significant (range 0.17–0.99) in the conditional analysis, suggesting they represent one signal (Table 1). The haplotype block containing the top SNP is within the non-coding region of Suppressor of Lin-12-Like Protein 1 (*SEL1L*) gene (Fig. 2). Having at least one risk haplotype was substantially more common among TZL (29%) versus TZUS or controls (12 and 7.5%, respectively).

**Table 1:** SNPs significantly associated with TZL at the genome-wide level

SNP	Chr	BP	Alleles	Associated Allele Frequency		R^**2**^ from top SNP	GWAS results		Conditional GWAS with Chr8 top SNP		Conditional GWAS with Chr14 top SNP	
				TZL	Ref		OR	***P***-value	OR	***P***-value	OR	***P***-value
BICF2S23237035	2	77,686,623	T/C	0.11	0.05		1.41	6.21E-05				
BICF2P1011303	8	52,650,576	T/C	0.18	0.07	0.53	1.30	4.60E-05	1.03	0.663		
BICF2P29000	8	52,763,337	C/A	0.25	0.12	0.28	1.24	7.46E-05	1.07	0.170		
BICF2P378684	8	53,742,667	C/T	0.15	0.04	0.59	1.42	5.71E-06	1.04	0.560		
BICF2P1080535	8	53,778,185	T/C	0.16	0.05	0.75	1.36	1.50E-05	0.99	0.935		
BICF2P1048848	8	53,785,948	A/G	0.16	0.05	0.75	1.36	1.50E-05	0.99	0.935		
BICF2P184533	8	53,796,442	G/A	0.16	0.06	0.79	1.37	7.36E-06	1.00	0.978		
**BICF2P948919**	8	53,818,371	G/A	0.21	0.07	1.00	1.39	2.66E-07	1.00	1.000		
TIGRP2P186605	14	11,778,977	G/A	0.66	0.43	0.93	1.18	5.13E-05			1.00	0.995
BICF2G630521678	14	11,791,385	A/G	0.67	0.43	0.99	1.18	3.00E-05			1.00	0.966
**BICF2G630521681**	14	11,794,735	C/T	0.67	0.43	1.00	1.19	2.28E-05			1.00	1.000
BICF2G630521696	14	11,807,161	G/A	0.67	0.43	0.99	1.18	3.67E-05			1.00	0.934
BICF2S23029378	17	4,217,272	G/A	0.88	0.73		1.19	9.47E-05				
BICF2G630222435	17	8,102,574	T/G	0.83	0.64		1.18	8.66E-05				
BICF2P916139	17	8,135,932	T/A	0.82	0.63		1.18	8.39E-05				
BICF2G630221951	17	8,819,612	C/T	0.85	0.65		1.19	9.42E-05				
BICF2P780894	29	10,587,617	G/A	0.63	0.46		1.18	7.45E-05				

The top SNPs (smallest *p-*value) for the chromosome 8 and 14 peaks are bolded. Ref represents the combined TZUS and control reference group

### Targeted resequencing of the chromosome 8 region identifies potential regulatory variants

Targeted resequencing was performed on 16 dogs selected for variation in risk and non-risk haplotypes. Sequence capture of the 3 Mb region on chromosome 8 identified 814 single nucleotide variants (SNVs) and 229 insertions and deletions (indels) that passed our filters. Median coverage across the region was 131x. Three synonymous coding variants were found in the *SEL1L* gene (cfa8:53,771,782, cfa8:52,779,502, cfa8:53,797,623). All other identified variants were potential modifiers, including 3′ UTR variants (three SNVs and one indel near *CEP128*, two SNVs near *GTF2A1*), up- and downstream gene variants, intron variants, and non-coding transcript exon variants (Additional files 2 and 3). Evaluation of the corresponding positions in the human genome determined multiple variants were in potential regulatory elements (of 685 that were converted [541 SNV, 144 indels]; based on H3K27AC marks and GeneHancer scoring). Two sets of variants were in enhancers for *DIO2* (Type II Iodothyronine Deiodinase) and seven sets of variants were in enhancers for combinations of *CEP128* (Centrosomal Protein 128), *GTF2A1* (General Transcription Factor IIA Subunit 1), *STON2* (Stonin2), and *SEL1L* (Additional file 4).

### Shared association with mast cell tumor cases on chromosome 14

**Fig. 3:**
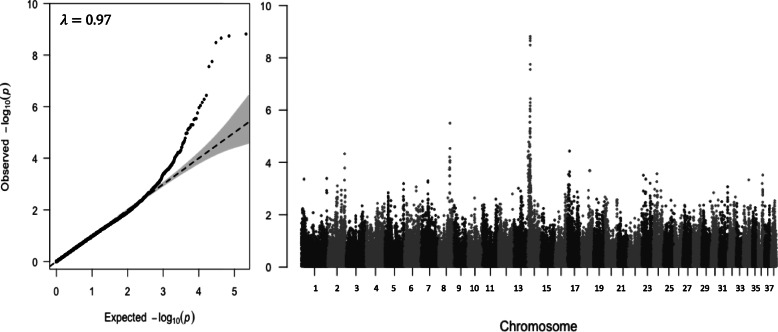
GWA for combined TZL and MCT datasets. QQ-plot (left) and Manhattan plot (right)

**Fig. 4:**
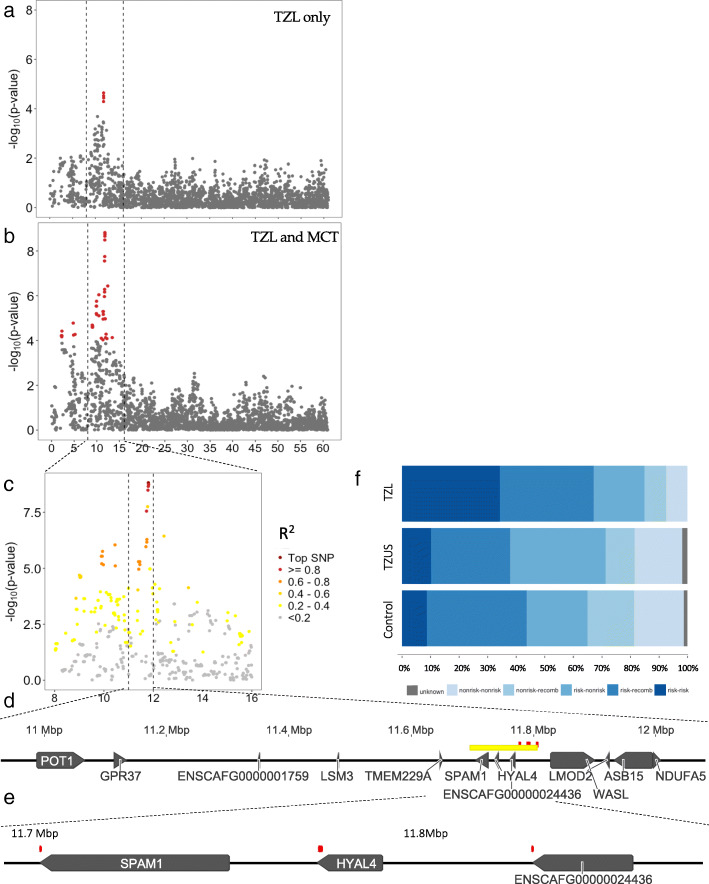
Close-up of the chromosome 14 peak depicting change in signal with MCT dataset added. **a** TZL dataset only. **b** Combined TZL and MCT dataset; **c** Close-up of the region from 8-12Mbp containing SNPs with R^2^ > 0.2. **d** Close-up view of genes located in the region from 11 to 12 Mb. The four SNPs significantly associated with TZL are depicted in red and the nine-SNP haplotype block they represent is shaded in yellow. **e** Close-up of the region from 11.7–11.8 Mbp where coding mutations (shown in red) were found on resequencing. **f** Haplotype block containing nine associated SNPs on cfa14 (BICF2G630521558, BICF2G630521572, BICF2G630521606, BICF2G630521619, BICF2P867665, TIGRP2P186605, BICF2G630521678, BICF2G630521681, BICF2G630521696). The risk haplotype was CTTCGGACG and non-risk was TCCTTAGTA. Dogs were considered recombined if neither combination was present and were considered unknown if the genotype for one or more SNPs was missing

The second top association peak is on chromosome 14 and contains four SNPs from 11,778,977–11,807,161 bp (Table 1). All SNPs were in strong LD (R^2^ > 0.9) with the top SNP (OR = 1.18, *p* = 8.39 × 10^− 5^). Three of the four SNPs had previously been reported to be associated with mast cell tumors (MCTs) among American Golden Retrievers [10]. Thus, we assessed our data in combination with the American Golden Retriever data from the publicly available MCT dataset.^1^ After independently conducting the quality control protocol outlined in the methods section for each dataset, files were merged so that the new “case” population included TZL and MCT cases, whereas the reference population contained TZUS and controls from the TZL dataset and controls from the MCT dataset. Multidimensional scaling (MDS) was performed using PLINK to assess for population stratification (Additional file 5). The chromosome 14 peak for the combined dataset was wider and more strongly associated, with the top SNP reaching *p* = 1.5 × 10^− 9^ (Fig. 3; similar association shown in Additional file 6A without the addition of controls from the MCT dataset). A GWAS including the TZL dataset and only MCT controls showed no increased association at the chromosome 14 peak (Additional file 6B), confirming that this is a shared association for the two different cancers and not simply a result of increased power from the additional controls. We evaluated haplotype blocks in the combined dataset. The top SNP from the combined dataset was the same as the top SNP in the TZL-only dataset (BICF2G630521681; Table 2). These SNPs are part of a nine SNP haplotype block that spans 11,695,969–11,807,161 bp (Fig. 4). When we ran a conditional GWAS controlling for the top SNP, none of the SNPs in the larger associated region remained significant (*p* > 0.3), suggesting they all represent one signal (Table 2, Additional file 7). The haplotype block containing the top SNP spans three hyaluronidase enzymes, including Sperm Adhesion Molecule 1 (*SPAM1*; formerly called *HYAL1*), Hyaluronoglucosaminidase 4 (*HYAL4*), and a hyaluronidase 4-like gene (*ENSCAFG00000024436*/*HYALP1*). In our dataset, 85% of TZL cases (67/79) had at least one risk haplotype (versus 71% of TZUS [77/108] and 65% of controls [52/80]); 34% of TZL were homozygous (27/79) for the risk haplotype (versus 7% of TZUS [11/108] and 9% of controls [7/80]) (Fig. 4).

**Table 2:** SNPs in the chromosome 14 haplotype block from the combined TZL + MCT GWAS

SNP	Chr	BP	Alleles	Associated Allele Freq		R^**2**^ from top SNP	TZL + MCT GWAS		Conditional for Chr14	
				TZL	Ref		OR	***P***-value	OR	***P***-value
BICF2G630521558	14	11,695,969	C/T	0.78	0.59	0.62	1.19	1.09E-06	1.00	0.9482
BICF2G630521572	14	11,721,433	T/C	0.70	0.45	0.94	1.22	2.80E-08	0.99	0.7588
BICF2G630521606	14	11,733,161	T/C	0.78	0.58	0.61	1.19	6.84E-07	1.01	0.8209
BICF2G630521619	14	11,736,615	C/T	0.79	0.58	0.60	1.19	5.17E-07	1.01	0.7721
BICF2P867665	14	11,765,081	G/T	0.79	0.57	0.59	1.22	1.77E-08	1.03	0.3593
**TIGRP2P186605**	14	11,778,977	G/A	0.70	0.43	0.95	1.23	3.25E-09	1.00	0.9681
**BICF2G630521678**	14	11,791,385	A/G	0.70	0.44	0.99	1.23	2.21E-09	1.00	0.9637
**BICF2G630521681**	14	11,794,735	C/T	0.70	0.43	1.00	1.23	1.51E-09	1.00	1.0000
**BICF2G630521696**	14	11,807,161	G/A	0.71	0.44	0.98	1.23	1.79E-09	1.00	0.9863

Only SNPs that are part of the nine-SNP haplotype block are shown. All SNPs that formed the chromosome 14 peak in the combined TZL + MCT GWAS are shown in Additional File 7. SNPs that were significant in the TZL only GWAS are bolded. The top SNP (BICF2G630521681) was the same for both analyses. Ref represents the combined TZUS and control reference group

### Targeted resequencing of chromosome 14 region identifies coding mutations in hyaluronidase genes

Median coverage across the 8 Mb region sequenced on chromosome 14 was 140x; 1404 SNVs and 742 indels were identified after quality control and filtering. Five mutations causing amino acid changes within coding regions of the three hyaluronidase genes (*SPAM1*, *HYAL4*, and *ENSCAFG00000024436*) were identified (Fig. 4); all mutations followed the associated haplotype identified by GWAS. The mutation within the *SPAM1* gene (cfa14:11,704,952, Lys482Arg) was predicted to be “possibly damaging” (PolyPhen-2 score 0.91). The three mutations in the *HYAL4* gene (cfa14:11,736,613, Gly454Ser; cfa14:11,736,674, Ser434Phe; cfa14:11,736,843, Leu378Ile) and one within *ENSCAFG00000024436* (cfa14:11,760,826, Met463Thr) were predicted to be benign (PolyPhen-2 score < 0.15). Conversion of these coordinates to CanFam2 determined the non-synonymous mutations in *SPAM1* and *HYAL4* were identical to those identified in the MCT study [10]. Additional non-coding variants were identified near these genes, including 5′ UTR variants (two SNVs, one indel in *HYAL4*), 3′ UTR variants (two SNVs, two indels in *HYAL4* and three SNVs in *SPAM1*), up- and downstream gene variants, and intron variants (Additional files 2 and 3). One synonymous coding SNP was identified in *ENSCAFG00000024436* (cfa14:11,768,664) (Additional file 2).

### Potential cumulative risk for chromosomes 8 and 14

**Fig. 5:**
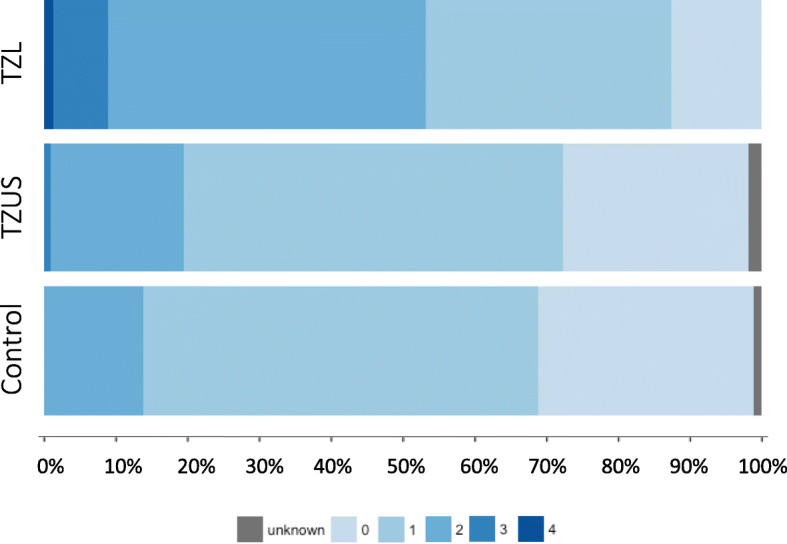
Distribution of haplotype scores. Dogs were scored from zero to four based on the number of risk haplotypes for chromosomes 8 and 14. Recombined haplotypes were considered non-risk. Dogs were considered unknown if the genotype for one or more SNPs was missing

Distribution of number of risk haplotypes by phenotype are shown in Fig. 5. Only 8 dogs (7 of which were cases) had > 3 risk haplotypes, so counts were categorized as 0, 1, > 2 for analysis. Number of risk haplotypes was significantly associated with TZL (*p*-value < 0.001), indicating a potential cumulative risk. Larger sample sizes are necessary to evaluate statistical interaction of the chromosome 8 and 14 haplotypes.

### Additional significantly associated GWAS SNPs

Associated SNPs were also seen on chromosomes 2, 17, and 29, but our study did not have the power to accurately determine the regions of association. We conducted a restricted maximum likelihood analysis [11], assuming TZL has a 2% prevalence in the Golden Retriever breed, and found that the combined set of 17 significant SNPs in our dataset (Table 1) explained approximately 15% (standard error 7%) of the phenotypic variance, whereas all genotyped SNPs explained approximately 49% (standard error 13%).

## Discussion

In a GWAS to identify genetic risk factors for TZL in Golden Retrievers, we identified associated regions on chromosomes 8 and 14. Subsequent resequencing of a subset of dogs identified non-synonymous mutations in three hyaluronidase genes on chromosome 14 (*SPAM1, HYAL4,* and *HYALP1)*. Coding mutations were not found in the chromosome 8 region but identified variants may be located in regulatory elements for numerous genes, including *DIO2, CEP128, GTF2A1, STON2*, and *SEL1L*.

### Mutations in hyaluronidase genes are associated with risk for TZL and MCT

GWAS analysis and subsequent resequencing identified mutations in *SPAM1* and *HYAL4* identical to those seen in Arendt et al.’s MCT study [10], highlighting a potential shared mechanism for TZL and MCT pathogenesis. One potential mechanism is via hyaluronan turnover, which is caused by the interaction of hyaluronan and CD44, a cell surface glycoprotein expressed on both T cells and mast cells [12]. This turnover leads to increased low molecular weight hyaluronan, the byproducts of which are pro-inflammatory and pro-oncogenic, with implications in cell proliferation, migration, and angiogenesis [13, 14]. In contrast, high molecular weight hyaluronan and decreased hyaluronidase activity have been associated with the increased longevity and cancer resistance seen in naked mole rats [13]. It would be informative to measure hyaluronan in TZL and controls to determine whether the ratio of low to high molecular weight hyaluronan is altered in TZL.

Most mammals have six hyaluronidase-like genes, clustered on two chromosomes. In dogs, *HYAL1*, *HYAL2* and *HYAL3* are clustered on cfa20, whereas *SPAM1*, *HYAL4*, and *ENSCAFG00000024436* are clustered on cfa14. *ENSCAFG00000024436* is homologous to *HYALP1*, which is an expressed pseudogene in people [15]. *HYALP1* is believed to be functional in other mammals [15], although its functional status is unknown in dogs. *SPAM1* is considered a testis hyaluronidase and is important during egg fertilization by sperm [16]. However, *SPAM1* has been detected in the epididymis, seminal vesicles, prostate, female genital tract, breast, placenta, fetal tissue, and certain malignancies [17–19], suggesting it is multifunctional and not sperm-specific. Despite the potential shared pathogenesis of TZL and MCT, we did not see an association between MCT and TZL in our dataset. Of dogs where medical history was known, 3/76 TZL (4%), 8/142 TZUS (6%), and 4/103 controls (4%) had a history of or concurrent MCT. This suggests these the diseases develop independently despite their shared mechanisms. More research is necessary to understand the role of these hyaluronidases in dogs and evaluate how the observed variants alter expression of hyaluronidases and downstream signaling.

### Thyroid hormone metabolism may influence TZL risk

In a parallel study, we determined dogs with hypothyroidism were significantly less likely to develop TZL than dogs without hypothyroidism [9]. As thyroid hormone plays an important role in cell growth and metabolism, we hypothesize that lack of this hormone may decrease T cell proliferation and therefore help prevent the development of TZL. A recent study reported an association between polymorphisms in *CEP128* and autoimmune thyroid disease in humans, although the mechanism underlying this association is unclear [20]. While we did not identify coding mutations within *CEP128*, it is possible that mutations we identified in regulatory elements could have similar downstream effects. Additionally, while SnpEff did not predict our SNVs to be modifiers of *DIO2* or *TSHR*, it is possible that the regulatory elements of these genes are far up- or downstream as seen in people. Canine genome annotations for this region may not yet be able to predict these relationships.

While canine hypothyroidism is generally thought to be caused by lymphocytic thyroiditis or idiopathic atrophy [21], it is plausible that changes in expression of *DIO2* or *TSHR* could influence its development. Thyroid hormone regulation depends on an axis of multiple hormones and organs. Thyroid stimulating hormone, released from the pituitary, binds *TSHR* on the thyroid gland, causing release of thyroxine and, to a lesser extent, triiodothyronine [22]. *DIO2* is one of two hormones responsible for converting thyroxine to triiodothyronine, the more active form, in the peripheral organs [23]. It is feasible that changes in the expression of either of these genes could alter thyroid hormone production and function. *SEL1L*, another gene in this region, encodes a protein that is part of a complex involved in endoplasmic reticulum-associated degradation of misfolded proteins [24]. Interestingly, levels of Deiodinase 2, the product of *DIO2*, are tightly regulated, and synthesis can be inhibited by endoplasmic reticulum stress via endoplasmic reticulum-associated degradation [25]. Thus, alterations in *SEL1L* and endoplasmic reticulum stress could also impact thyroid hormone regulation. While a role of thyroid hormone is plausible based on variants identified on cfa8 and our parallel finding of an inverse association of hypothyroidism and TZL risk, we cannot rule out the possibility of a spurious finding due to chance overrepresentation of dogs with hypothyroidism among our control population. Further studies are needed to validate this finding in an independent population.

### Golden Retriever predisposition to TZL

We believe TZUS represents a precursor state to TZL, so we hypothesized TZUS dogs would share the same genetic variants as TZL cases. However, we were unable to differentiate TZUS from controls in our GWAS analysis. The high prevalence of TZL among Golden Retrievers and corresponding high prevalence of TZUS suggest the genetic basis for developing CD5^+^CD45^−^ T cells may be fixed among this breed, and different from the genes that control progression to neoplasia in these cells. If this is the case, we would be unable to identify the genetic risk factor for developing CD5^+^CD45^−^ T cells in our study. Future studies may evaluate fixed regions of the Golden Retriever genome to identify candidate genes. Additionally, a GWAS of TZL among a less predisposed breed may distinguish additional associated regions not identified in our study. It is worth noting that Golden Retrievers of European descent appear less likely to develop TZL [26]. As such, delving into genomic differences between European and American Golden Retrievers may provide insight into regions that could underly TZL risk.

## Conclusions

Canine genomics are informative for human genomics and offer computational benefits due to the comparatively recent development of dog breeds. Within a dog breed, there is reduced genetic variation [27, 28], allowing us to use smaller sample sizes and fewer genetic markers when evaluating genetic risk factors for canine diseases [28–30]. Little is known about the functional implications of the mutations identified on cfa8. Since variants in this region are in moderate to high LD, it is difficult to prioritize which variants are important in disease pathogenesis versus which are bystanders inherited with the causative mutation. Additional studies are necessary to elucidate these associations and better understand the effect of these variants. The likely importance of hyaluronidases and shared association with MCT is noteworthy and warrants further investigation. Further research will increase our understanding of how these coding mutations alter hyaluronidase function. Ultimately, future research will help elucidate TZL pathogenesis and identify causative variants that may be biomarkers or disease risk or potential therapeutic targets.

## Methods

### Study participants

All dogs were recruited from the privately-owned pet population in the United States from October 2013 through May 2015. The study was conducted with approval from the Colorado State University Institutional Animal Care and Use committee (Protocol 13-4473A). Written informed consent was obtained from all dog owners; dogs remained under the custody and care of their owners for the duration of the study. Detailed recruitment information for the larger study population has been previously described [9]. Briefly, TZL cases were identified through submissions to Colorado State University’s Clinical Immunology laboratory. Lymphoma-free Golden Retrievers aged > 9 years were recruited from 1) the submitting clinic of TZL cases and 2) email solicitation to Golden Retriever owners in the Canine Lifetime Health Project [9]. Peripheral blood samples were obtained from all participants, and a subset of dogs with adequate DNA quality and quantity (as described below) were used for the GWAS study. Flow cytometric analysis of peripheral blood samples was used to categorize dogs as TZL, TZUS, or controls. Flow cytometry was carried out as previously described [3] and samples were analyzed with the antibody combinations listed in Additional file 8 using a 3-laser Coulter Gallios.^2^ We defined TZL cases (*n* = 95) as a homogeneous expansion of CD5^+^CD45^−^ T cells and lymphocytosis (> 5000 lymphocytes/μL), lymphadenopathy (noted on veterinarian-completed submission form), or both (Additional file 9A). We defined dogs as TZUS (*n* = 142) if they were > 9 years of age and had no history or clinical signs of a lymphoproliferative disease (no lymphadenopathy or lymphocytosis), but had a small population of CD5^+^CD45^−^ T cells on flow cytometry (> 1% of total T cells; Additional file 9B). Control dogs (*n* = 101) were those > 9 years of age with no history or suspicion of a lymphoproliferative disease, no population of CD5^+^CD45^−^ T cells identified by flow cytometry (< 1% of total T cells; Additional file 9C), and no evidence of a clonal T cell population in the peripheral blood as assessed using the PCR for Antigen Receptor Rearrangement (PARR) assay [31].

### Genome-wide association mapping

Genomic DNA was extracted from white blood cell pellets of peripheral blood samples using the QIAamp DNA blood Midi Kit.^3^ DNA quality and quantity were assessed using NanoDrop^4^ and only samples with 1) a concentration of at least 35 ng/μL, 2) over 1000 ng of DNA total, and 3) an A260/280 ratio of 1.7–2.0 were submitted for genotyping. Genotyping was performed at GeneSeek Inc.^5^ using the Illumina 170 K CanineHD BeadChip SNP array [32]. PLINK software [33, 34] was used to perform data quality control, removing individuals with call rates < 97.5% and SNPs with call rates < 97.5% or minor allele frequency < 5%. Only autosomal chromosomes were analyzed.

MDS was performed using PLINK to ensure there were no distinct groupings based on phenotype (TZL/TZUS/control), which would indicate population stratification and/or residual confounding. While we saw no obvious deviations on these plots, we were concerned for bias based on European versus American descent due to apparent divergence in this breed [10, 29]. To determine whether any of our dogs were likely of European descent, we downloaded a publicly available dataset^1^ [10] including both European and American Golden Retrievers, conducted the same quality control protocol as described above, and merged the two datasets. We then created MDS plots to determine which dogs in our dataset clustered with the known European dogs. Dogs with a value for the first cluster < 2.5 standard deviations from the mean value for known European dogs were removed (Additional file 10).

Genome-wide complex trait analysis (GCTA) software [11] was used to estimate a genetic relationship matrix (GRM) and remove highly related individuals (one dog was removed for each pair of dogs with the same phenotype and a GRM value of 0.25 [half-sibling level]). The disease-genotype association was estimated using GCTA, adjusting for the first principal component of the GRM in a mixed linear model to correct for cryptic relatedness [35]. QQ-plots with 95% CIs calculated based on the beta distribution of observed *p*-values were created to assess possible genomic inflation and to establish suggestive significance levels.

It is currently unclear whether TZUS dogs share some or all of the genetic predisposition for TZL, or they simply represent normal variation among controls. To determine in which category they are most genetically informative, we performed separate association studies comparing 1) combined TZL and TZUS versus controls, 2) TZL versus combined TZUS and controls, and 3) pairwise comparisons (TZL versus control, TZL versus TZUS, and TZUS versus control). Quantile-quantile plots were used to determine which analyses had enough power to be evaluated. After peaks of interest were identified, we used GCTA to conduct a conditional GWAS. By adjusting for the genotype of the top SNP of each peak of interest, we can evaluate whether the other significantly associated SNPs in that peak are statistically independent from the top SNP (i.e. whether there are one or multiple peaks).

### Haplotype block definition and association analysis

Haplotype blocks for associated loci were defined based on boundaries identified both by clumping analysis in PLINK and R^2^-based LD analysis in Haploview [36]. For clumping analysis, the dataset was subsetted to a region including all SNPs with R^2^ > 0.2 from the top SNP on that chromosome. Because of the genomic structure of dogs, a maximum window size was set to 5000 kb. For each block, their haplotype, frequencies, chi-square test, and *p*-value were obtained using PLINK. To assess cumulative risk, we additionally categorized dogs by number of risk haplotypes (zero to four) present for the associated regions. Logistic regression was used to evaluate the association of number of risk haplotypes and TZL.

### Targeted sequencing

Sixteen dogs (10 TZL, 3 TZUS, 3 controls), selected for optimal haplotype representation (i.e. to represent risk–risk, risk–non-risk, and non-risk–non-risk for each haplotype) and distribution in MDS plot, were sequenced across the associated genomic regions (Additional file 11). A custom sequence capture array was designed (NimbleGen SeqCap EZ Developer Kit^6^) to cover the top associated regions (16.1 Mb total; CanFam 3.1 cfa8:51,700,000-54,800,000, cfa14:8,000,000-16,100,000, cfa29:7,600,000-12,500,000). Regions were chosen to include all SNPs with R^2^ > 0.2 from the top SNP. Standard indexed Illumina libraries were prepared with the KAPA HyperPlus library preparation kit.^7^ Targeted pooled (4 samples) libraries were captured by hybridization in solution using the custom probe pool. Estimated coverage of the 16.1 Mb target region was 95%. Library constructions, pooling and captures were performed following the SeqCap EZ HyperCap Workflow User’s Guide (V 1.0)^6^. Per suggestion from NimbleGen, developer’s reagent (06684335001) was used in place of COT-1. Index-specific hybridization enhancing oligonucleotides were used to improve the efficiency of genomic region capture. Sequencing was carried out on an Illumina NextSeq 500.

Sequencing data were pre-processed and aligned to the CanFam3.1 reference genome^8^ using FastQC [37], Samtools [38, 39], Picard Tools [40], Genome Analysis Toolkit [41], and BWA-MEM [42], as specified by Genome Analysis Toolkit best practices. Data was visualized using Integrative Genomics Viewer [43]. Genome Analysis Toolkit was used for base quality score recalibration (BaseRecalibrator), variant calling (HaplotypeCaller), and variant prioritization (VariantFiltration). Variants were additionally filtered based on adherence to the risk or non-risk haplotype (at least 80% adherence) and variants that passed this filter were annotated using SnpEff [44]. Coding SNPs were evaluated for predicted effect using PolyPhen-2 [45]. Ensembl was used to convert coordinates to the human genome to determine whether non-coding SNPs were in potential regulatory elements.

## Supplementary information

**Additional file 1.** GWA for all TZL combinations. QQ-plot (left) and Manhattan plot (right). A) TZL case + TZUS vs. control; B) TZL case vs. control; C) TZL case vs. TZUS.

**Additional file 2.** Summary of snpEff annotations for single nucleotide variants.

**Additional file 3.** Summary of snpEff annotations for indels.

**Additional file 4.** Summary of potential regulatory elements.

**Additional file 5.** Multidimensional scaling plot for combined TZL and MCT datasets. Plot is colored by phenotype and dataset.

**Additional file 6.** GWA for combined TZL and MCT datasets. QQ-plot (left) and Manhattan plot (right). A) GWAS of combined TZL and MCT cases versus TZUS and TZL controls; B) GWAS of TZL cases versus combined TZUS, TZL controls, and MCT controls.

**Additional file 7.** SNPs in the chromosome 14 peak from the combined TZL + MCT GWAS.

**Additional file 8.** Antibody panels used for immunophenotyping.

**Additional file 9.** Flow cytometric analysis of peripheral blood samples. (A) Sample considered diagnostic for TZL due to homogeneous expansion of CD5^+^CD45^−^ T cells (red cells). (B) Sample diagnosed as TZUS due to smaller population of CD5^+^CD45^−^ T cells and absence of lymphocytosis or lymphadenopathy. (C) Sample considered a control; all T cells are CD5 + CD45+ (green cells; normal).

**Additional file 10.** Multidimensional scaling plot showing clustering of European dogs. Dogs marked in red met our threshold for potential European origin based on clustering and were subsequently removed from the analysis.

**Additional file 11.** Signalment, GWAS haplotype, and coverage for 16 resequenced dogs.

## Data Availability

The datasets analysed during the current study are available at 10.25675/10217/208318 (primary data collected for this study), https://www.broadinstitute.org/ftp/pub/vgb/dog/MCT_GWAS_PLOSGenetics_2015/ (data from Arendt et al. MCT GWAS), https://www.ncbi.nlm.nih.gov/assembly/GCF_000002285.3/ (CanFam 3.1 reference genome).

## Abbreviations

CI: Confidence interval
GCTA: Genome-wide complex trait analysis
GRM: Genetic relationship matrix
GWAS: Genome-wide association study
LD: Linkage disequilibrium
MCT: Mast cell tumor
MDS: Multidimensional scaling
OR: Odds ratio
PTCL: Peripheral T cell lymphoma
SNP: Single nucleotide polymorphism
SNV: Single nucleotide variant
TZL: T zone lymphoma
TZUS: T zone-like cells of undetermined significance
QQ: Quantile-quantile

## Footnotes

1 Genotyping data are available on the BROAD website: https://www.broadinstitute.org/ftp/pub/vgb/dog/MCT_GWAS_PLOSGenetics_2015/

2 Beckman Coulter Inc., Brea, CA

3 QIAGEN Inc., Germantown, MD

4 Thermo Scientific, Wilmington, DE

5 GeneSeek Inc., Lincoln, NE

6 Roche Diagnostics Corporation, Indianapolis, IN

7 KapaBiosystems, Wilmington, MA

8 CanFam3.1 reference genome is available at: https://www.ncbi.nlm.nih.gov/assembly/GCF_000002285.3/

## References

[1] Ito D, Frantz AM, Modiano JF (2014). Canine lymphoma as a comparative model for human non-Hodgkin lymphoma: recent progress and applications. Vet Immunol Immunopathol.

[2] Valli V (2007). Mature (peripheral) nodal T-cell (T-zone) lymphoma. Veterinary Comparative Hematopathology.

[3] Seelig DM, Avery P, Webb T, Yoshimoto J, Bromberek J, Ehrhart EJ (2014). Canine T-zone lymphoma: unique immunophenotypic features, outcome, and population characteristics. J Vet Intern Med.

[4] Flood-Knapik KE, Durham AC, Gregor TP, Sánchez MD, Durney ME, Sorenmo KU. Clinical, histopathological and immunohistochemical characterization of canine indolent lymphoma. Vet Comp Oncol. 2012. 10.1111/j.1476-5829.2011.00317.x 22296667

[5] Valli VE, Vernau W, de Lorimier LP, Graham PS, Moore PF (2006). Canine indolent nodular lymphoma. Vet Pathol.

[6] Valli VE, San Myint M, Barthel A, Bienzle D, Caswell J, Colbatzky F (2011). Classification of canine malignant lymphomas according to the World Health Organization criteria. Vet Pathol.

[7] Mizutani N, Goto-Koshino Y, Takahashi M, Uchida K, Tsujimoto H (2016). Clinical and histopathological evaluation of 16 dogs with T-zone lymphoma. J Vet Med Sci.

[8] Hughes KL, Labadie JD, Yoshimoto JA, Dossey JJ, Burnett RC, Avery AC. Increased frequency of CD45 negative T cells (T zone cells) in older Golden retriever dogs. Vet Comp Oncol. 2017. 10.1111/vco.12343 28905476

[9] Labadie JD, Magzamen S, Morley PS, Anderson GB, Yoshimoto J, Avery AC (2019). Associations of environment, health history, T-zone lymphoma, and T-zone-like cells of undetermined significance: a case-control study of aged Golden retrievers. J Vet Intern Med.

[10] Arendt ML, Melin M, Tonomura N, Koltookian M, Courtay-Cahen C, Flindall N (2015). Genome-wide association study of Golden retrievers identifies germ-line risk factors predisposing to mast cell Tumours. PLoS Genet.

[11] Yang J, Lee SH, Goddard ME, Visscher PM (2011). GCTA: a tool for genome-wide complex trait analysis. Am J Hum Genet.

[12] Toole BP (2001). Hyaluronan in morphogenesis. Semin Cell Dev Biol.

[13] Tian X, Azpurua J, Hine C, Vaidya A, Myakishev-Rempel M, Ablaeva J (2013). High-molecular-mass hyaluronan mediates the cancer resistance of the naked mole rat. Nature..

[14] Girish KS, Kemparaju K (2007). The magic glue hyaluronan and its eraser hyaluronidase: a biological overview. Life Sci.

[15] Csóka AB, Scherer SW, Stern R (1999). Expression analysis of six paralogous human hyaluronidase genes clustered on chromosomes 3p21 and 7q31. Genomics..

[16] Cherr GN, Yudin AI, Overstreet JW (2001). The dual functions of GPI-anchored PH-20: hyaluronidase and intracellular signaling. Matrix Biol.

[17] Zhang H, Martin-DeLeon PA (2003). Mouse Spam1 (PH-20) is a multifunctional protein: evidence for its expression in the female reproductive tract. Biol Reprod.

[18] Oettl M, Hoechstetter J, Asen I, Bernhardt G, Buschauer A (2003). Comparative characterization of bovine testicular hyaluronidase and a hyaluronate lyase from Streptococcus agalactiae in pharmaceutical preparations. Eur J Pharm Sci.

[19] Beech DJ, Madan AK, Deng N (2002). Expression of PH-20 in normal and neoplastic breast tissue. J Surg Res.

[20] Wang B, Jia X, Yao Q, Li Q, He W, Li L (2019). CEP128 is a crucial risk locus for autoimmune thyroid diseases. Mol Cell Endocrinol.

[21] Graham PA, Refsal KR, Nachreiner RF (2007). Etiopathologic findings of canine hypothyroidism. Vet Clin North Am Small Anim Pract.

[22] Dietrich JW, Landgrafe G, Fotiadou EH (2012). TSH and Thyrotropic agonists: key actors in thyroid homeostasis. J Thyroid Res.

[23] Gereben B, Zavacki AM, Ribich S, Kim BW, Huang SA, Simonides WS (2008). Cellular and molecular basis of deiodinase-regulated thyroid hormone signaling. Endocr Rev.

[24] Olzmann JA, Kopito RR, Christianson JC. The mammalian endoplasmic reticulum-associated degradation system. Cold Spring Harb Perspect Biol. 2013;5(9). 10.1101/cshperspect.a013185 PMC3753711 23232094

[25] Arrojo E, Drigo R, Fonseca TL, Castillo M, Salathe M, Simovic G, Mohácsik P (2011). Endoplasmic reticulum stress decreases intracellular thyroid hormone activation via an eIF2a-mediated decrease in type 2 deiodinase synthesis. Mol Endocrinol.

[26] Comazzi S, Marelli S, Cozzi M, Rizzi R, Finotello R, Henriques J (2018). Breed-associated risks for developing canine lymphoma differ among countries: an European canine lymphoma network study. BMC Vet Res.

[27] Ostrander EA, Franklin H (2012). Epstein lecture. Both ends of the leash--the human links to good dogs with bad genes. N Engl J Med.

[28] Lindblad-Toh K, Wade CM, Mikkelsen TS, Karlsson EK, Jaffe DB, Kamal M (2005). Genome sequence, comparative analysis and haplotype structure of the domestic dog. Nature..

[29] Karlsson EK, Baranowska I, Wade CM, Salmon Hillbertz NH, Zody MC, Anderson N (2007). Efficient mapping of mendelian traits in dogs through genome-wide association. Nat Genet.

[30] Karlsson EK, Lindblad-Toh K (2008). Leader of the pack: gene mapping in dogs and other model organisms. Nat Rev Genet.

[31] Burnett RC, Vernau W, Modiano JF, Olver CS, Moore PF, Avery AC (2003). Diagnosis of canine lymphoid neoplasia using clonal rearrangements of antigen receptor genes. Vet Pathol.

[32] Vaysse A, Ratnakumar A, Derrien T, Axelsson E, Rosengren Pielberg G, Sigurdsson S (2011). Identification of genomic regions associated with phenotypic variation between dog breeds using selection mapping. PLoS Genet.

[33] Purcell S, Chang C. PLINK [1.9] [Available from: https://www.cog-genomics.org/plink2.

[34] Chang CC, Chow CC, Tellier LC, Vattikuti S, Purcell SM, Lee JJ (2015). Second-generation PLINK: rising to the challenge of larger and richer datasets. Gigascience..

[35] Thomson R, McWhirter R (2017). Adjusting for familial relatedness in the analysis of GWAS data. Methods Mol Biol.

[36] Barrett JC, Fry B, Maller J, Daly MJ (2005). Haploview: analysis and visualization of LD and haplotype maps. Bioinformatics..

[37] FastQC: a quality control tool for high throughput sequence data: Babraham Bioinformatics; [Available from: http://www.bioinformatics.bbsrc.ac.uk/projects/fastqc/.

[38] Li H, Handsaker B, Wysoker A, Fennell T, Ruan J, Homer N (2009). The sequence alignment/map format and SAMtools. Bioinformatics..

[39] Li H (2011). A statistical framework for SNP calling, mutation discovery, association mapping and population genetical parameter estimation from sequencing data. Bioinformatics..

[40] Picard [Available from: http://picard.sourceforge.net.

[41] McKenna A, Hanna M, Banks E, Sivachenko A, Cibulskis K, Kernytsky A (2010). The genome analysis toolkit: a MapReduce framework for analyzing next-generation DNA sequencing data. Genome Res.

[42] Li H (2013). Aligning sequence reads, clone sequences and assembly contigs with BWA-MEM. q-bioGN. arXiv.

[43] Robinson JT, Thorvaldsdóttir H, Winckler W, Guttman M, Lander ES, Getz G (2011). Integrative genomics viewer. Nat Biotechnol.

[44] Cingolani P, Platts A, lL W, Coon M, Nguyen T, Wang L (2012). A program for annotating and predicting the effects of single nucleotide polymorphisms, SnpEff: SNPs in the genome of Drosophila melanogaster strain w1118; iso-2; iso-3. Fly (Austin).

[45] Adzhubei IA, Schmidt S, Peshkin L, Ramensky VE, Gerasimova A, Bork P (2010). A method and server for predicting damaging missense mutations. Nat Methods.

