# Supplementary figures and images for: Genome-wide association analysis of canine T zone lymphoma identifies link to hypothyroidism and a shared association with mast-cell tumors
Source: BMC Genomics. 2020 Jul 6;21:464. doi: 10.1186/s12864-020-06872-9 (PMC7339439; doi:10.1186/s12864-020-06872-9)

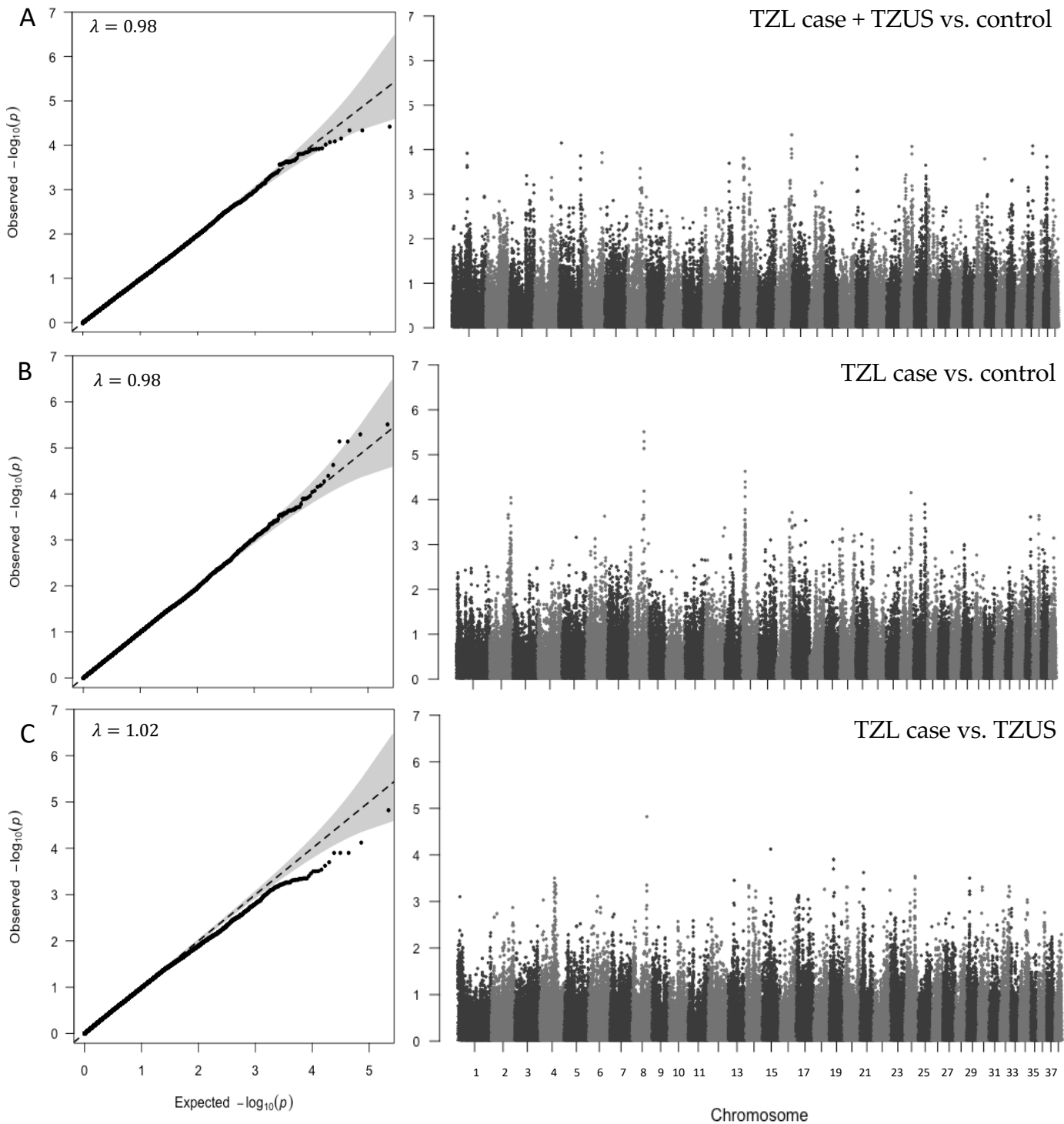

Supplement: Supplementary file 1 — Additional file 1. GWA for all TZL combinations. QQ-plot (left) and Manhattan plot (right). A) TZL case + TZUS vs. control; B) TZL case vs. control; C) TZL case vs. TZUS. [file 12864_2020_6872_MOESM1_ESM.pdf]

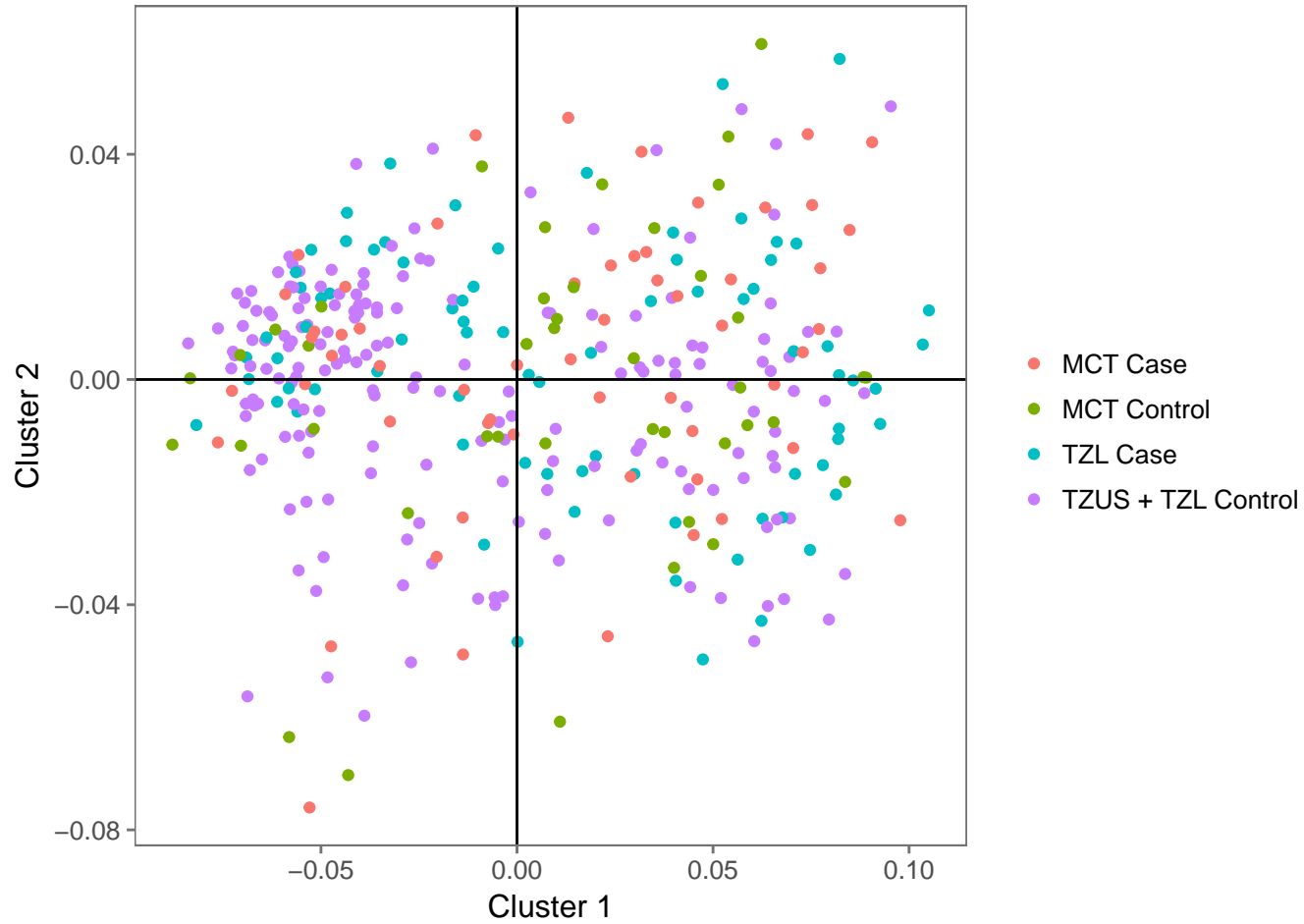

Supplement: Supplementary file 5 — Additional file 5. Multidimensional scaling plot for combined TZL and MCT datasets. Plot is colored by phenotype and dataset. [file 12864_2020_6872_MOESM5_ESM.pdf]

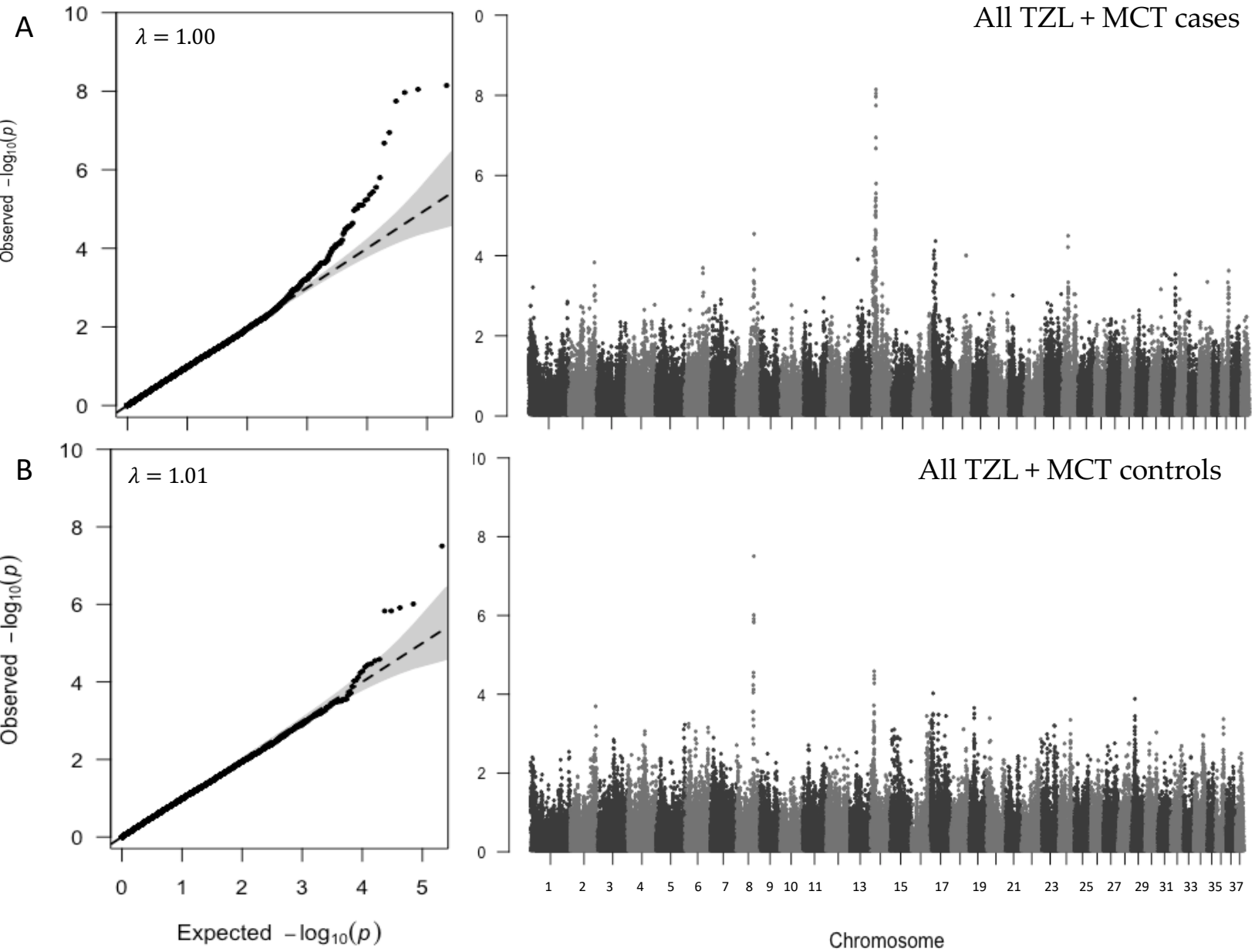

Supplement: Supplementary file 6 — Additional file 6. GWA for combined TZL and MCT datasets. QQ-plot (left) and Manhattan plot (right). A) GWAS of combined TZL and MCT cases versus TZUS and TZL controls; B) GWAS of TZL cases versus combined TZUS, TZL controls, and MCT controls. [file 12864_2020_6872_MOESM6_ESM.pdf]

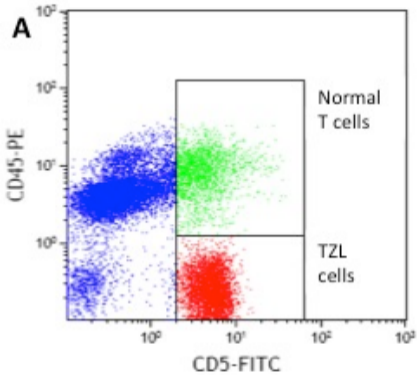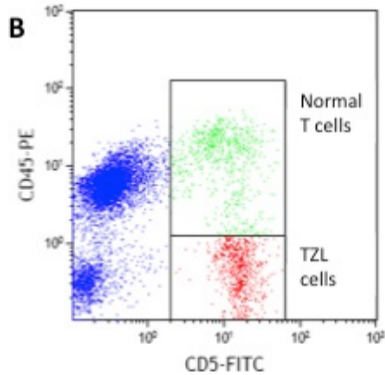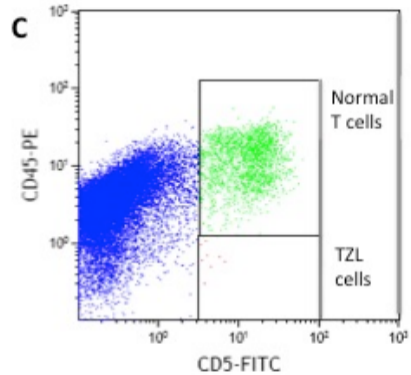

Supplement: Supplementary file 9 — Additional file 9. Flow cytometric analysis of peripheral blood samples. (A) Sample considered diagnostic for TZL due to homogeneous expansion of CD5+CD45− T cells (red cells). (B) Sample diagnosed as TZUS due to smaller population of CD5+CD45− T cells and absence of lymphocytosis or lymphadenopathy. (C) Sample considered a control; all T cells are CD5 + CD45+ (green cells; normal). [file 12864_2020_6872_MOESM9_ESM.pdf]

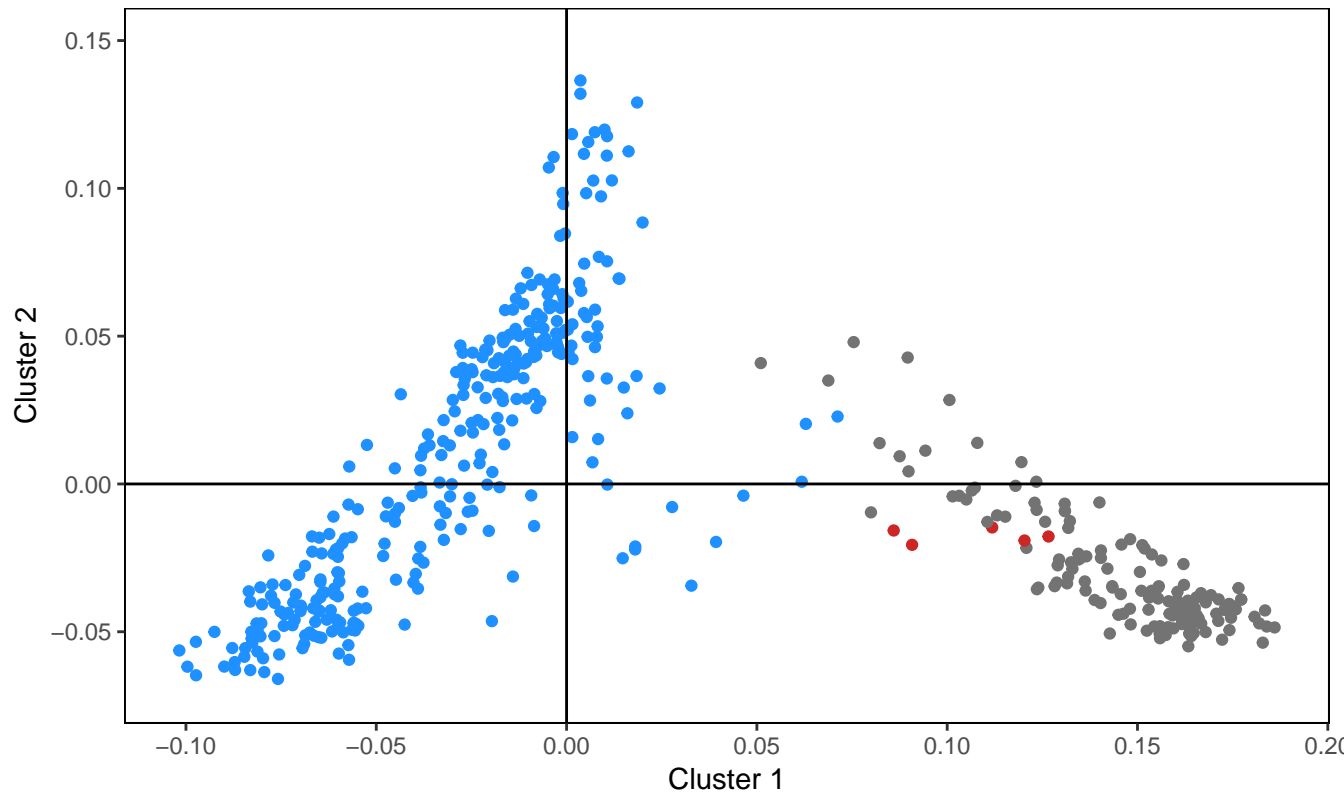

● Known European ● Potential European ● United States

Supplement: Supplementary file 10 — Additional file 10. Multidimensional scaling plot showing clustering of European dogs. Dogs marked in red met our threshold for potential European origin based on clustering and were subsequently removed from the analysis. [file 12864_2020_6872_MOESM10_ESM.pdf]
